# Supervised Pelvic Floor Muscle Training Improves Sexual Function and Diminishes Sexual Distress in Women with Relapsing–Remitting Multiple Sclerosis: A Randomised Controlled Study

**DOI:** 10.3390/jpm14010088

**Published:** 2024-01-12

**Authors:** Athanasios Zachariou, Athanasios Zikopoulos, Vaia Sapouna, Chara Skentou, Aris Kaltsas, Ioannis Giannakis, Dimitrios Zachariou, Fotios Dimitriadis, Charalampos Mamoulakis, Dung Ba Tien Mai, Nguyen Ho Vinh Phuoc, Atsushi Takenaka, Nikolaos Sofikitis

**Affiliations:** 1 Department of Urology, Faculty of Medicine, School of Health Sciences, University of Ioannina, 45110 Ioannina, Greece; kzikop22@gmail.com (A.Z.); johgiann@hotmail.com (I.G.); v.sofikitis@hotmail.com (N.S.); 2 Department of Urology, EV PRATTEIN Rehabilitation Centre, 38222 Volos, Greece; vsapouna@uth.gr (V.S.); dimitriszaxariou@yahoo.com (D.Z.); 3 Department of Obstetrics and, Gynecology, Faculty of Medicine, School of Health Sciences, University of Ioannina, 45500 Ioannina, Greece; haraskentou@uoi.gr; 4 Third Department of Urology, Attikon University Hospital, School of Medicine, National and Kapodistrian University of Athens, 12462 Athens, Greece; ares-kaltsas@hotmail.com; 5 Department of Urology, Faculty of Medicine, School of Health Sciences, Aristotle University of Thessaloniki, 54124 Thessaloniki, Greece; helabio@yahoo.gr; 6 Department of Urology, Faculty of Medicine, School of Health Sciences, University of Crete, 70013 Heraklion, Greece; mamoulak@uoc.gr; 7 Department of Andrology, Binh Dan Hospital, Ho Chi Minh City 70000, Vietnam; maibatiendung@yahoo.com (D.B.T.M.); nguyenhovinhphuoc@gmail.com (N.H.V.P.); 8 Department of Urology, Tottori University, Yonago 683-8503, Japan; atake@med.tottori-u.ac.jp

**Keywords:** pelvic floor muscle training, multiple sclerosis, sexual dysfunction, women’s health, randomised controlled trial, sexual function and distress

## Abstract

This study investigates the impact of pelvic floor muscle training (PFMT) on sexual function and distress in women with multiple sclerosis (MS), a prevalent chronic nervous system disorder associated with sexual dysfunction. This study’s primary aim was to assess the effectiveness of PFMT at improving sexual function and alleviating sexual distress in this population. In a randomised controlled trial, 82 women with MS were divided into two groups: Group A (41 women) underwent 12 weeks of PFMT, while Group B (41 women) served as a control group with no intervention. Both groups were assessed at the beginning and end of this study using the Female Sexual Function Index (FSFI) and Female Sexual Distress Scale-Revised (FSDS-R). Statistical analysis, including Chi-square tests, was employed to compare the outcomes between the two groups, with a *p*-value of less than 0.05 considered significant. The results revealed no significant differences in baseline sexual function and distress between the groups. However, at the conclusion of the 12-week period, Group A exhibited statistically significant improvements in nearly all domains of FSFI and FSDS-R compared to Group B, except in the pain domain. This study concludes that PFMT can effectively enhance sexual function and reduce sexual distress in women suffering from MS. These findings underscore the potential of PFMT as a therapeutic intervention in managing sexual dysfunction associated with MS.

## 1. Introduction

Female sexual disorders are a comparatively novel focus of bio-medical research. However, sexual dysfunction is actually more common in women (43%) than men and is correlated with several demographic characteristics, including educational status and age [1]. Female sexual dysfunction (FSD) deteriorates physical well-being and emotional health, as well as having a significant effect on the quality of life for women and their interpersonal relationships. Impaired sexual function can have harmful effects on self-esteem and cause decreased sexual pleasure, disruption of sexual activity and negative partner responses [2]. FSD is often emotionally distressing, and the consequences might lead to familial discordance and divorce [3].

FSD is a multifactorial and a multi–dimensional medical problem. Psychological, social, hormonal, neurological and environmental factors are considered paramount in shaping sexual function and behaviour. Merely focusing on psychological and emotional processes is insufficient when addressing women’s sexual function [4].

The most prevalent chronic neurological condition in young adults is multiple sclerosis (MS). It presents a wide range of potential consequences on neurological function, including the autonomic nervous system. In women with MS, FSD is a prevalent and extremely stressful condition [5]. According to Yasdani et al., FSD prevalence in MS patients was evaluated to be 61%, and the odds of presenting FSD in comparison with controls is 3.05 [6]. Reduced libido was described as the most reported issue, affecting 48% of patients. Furthermore, the pooled prevalence of satisfaction with intercourse was determined to be 27% [6]. MS patients present increased levels of physical disability, psychosocial variables and adverse pharmacological side effects, all of which can increase the incidence of FSD [7]. Sexual dysfunction can appear at different MS phases, beginning at an early stage of the illness [8] and becoming more prevalent as the disease progresses [9].

Recent studies have highlighted that bladder, bowel and sexual function problems have often been neglected in MS patients. These issues are prevalent in up to 75% of patients with MS, significantly affecting their quality of life [10]. Moreover, research indicates that sexual dysfunction levels, including primary, secondary and tertiary, are remarkably high in Iranian women suffering from MS, with cultural barriers playing a significant role in these findings [11].

Sexual dysfunctions in MS have been classified as primary, secondary or tertiary. Primary sexual dysfunction is a problem that results directly from neurological abnormalities brought on by MS. Lesions which demyelinate the central nervous system (CNS), for instance, can impair genital sensation, lower sexual desire, reduce vaginal lubrication and/or cause a decrease in the frequency and intensity of orgasms. Physical symptoms of MS are linked to secondary sexual dysfunction. For instance, spasticity, discomfort, pain, difficulty in mobility, bladder and bowel issues or fatigue may contribute to sexual dysfunction. The psychological, emotional, social, cultural, psychosexual and partnership-related aspects of MS that may lead to sexual discomfort are referred to as tertiary sexual dysfunction [12].

Pelvic floor muscles (PFM) play a role in improving female sexual function by generating involuntary rhythmic contractions during orgasm and heightening vaginal sensitivity during intercourse. Research findings suggest that over a third of MS patients present with signs of pelvic floor weakness [13]. Pelvic floor muscle training (PFMT) contributes to better sexual function, as PFM strength and the ability to properly experience improved vaginal receptivity and responsiveness, female orgasm and sexual pleasure [14]. Additionally, it is known that a weak pelvic floor reduces orgasm and arousal function, demonstrating the necessity of proper PFM functioning for sex life enjoyment [15].

Few studies present the effects of PFMT on the sexual function of MS patients without urinary incontinence [16,17]. A significant concern is that these studies present limitations, including the absence of female sexual distress evaluation. Understanding this is crucial because, based on diagnostic criteria for female sexual dysfunction, the disorder must lead to substantial distress in women.

Furthermore, investigations have found that specific pharmacological developments have been made in pro-erectile agents, which could potentially benefit women with MS experiencing sexual dysfunction [12].

The primary outcome of our study was to investigate the effects of supervised PFMT in terms of improving sexual function and sexual distress in women suffering from MS.

## 2. Materials and Methods

This study was a prospective randomised controlled study conducted from September 2021 to May 2023 at the EY PRATTEIN Rehabilitation Center. To estimate the sample size, an evaluation of a 95% confidence rate, an 80% power rate and a 10% dropout rate from the study groups resulted in a minimum number of 60 patients. Eighty-two women were finally recruited and consecutively randomised into two groups. The allocation sequence was concealed using sealed opaque envelopes to further ensure the impartiality of group assignment. Consecutive selection is a non-probability sampling technique where patients are selected at the ease of the investigator, more like a convenient sampling. This process warranted an unbiased and unpredictable assignment of patients into two groups, diminishing possible bias. The primary investigator enrolled participants and generated the random sequence into two groups.

Intervention Group A consisted of forty-one women suffering from MS and underwent PFMT for 12 weeks. Control Group B consisted of forty-one women with MS and formed the observation group (negative control group). Both groups received standard care, as per the centre’s protocol to maintain consistency in their treatment regimen. The institutional ethics committee approved this study (12/2021, 1 September 2021), and all female participants provided written consent before participation. Inclusion and exclusion criteria are presented in Table 1.

The stable condition of MS was a critical topic in the inclusion criteria to ensure consistency in the participant’s health status throughout this study. During the protocol period, women who exhibited worsening MS, double or blurred vision, increasing weakness of muscle, fatigue or loss of coordination were neurologically re-evaluated and given an EDSS score.

The Kurtzke Expanded Disability Status Scale (EDSS) ranges from 0 to 10. The initial groups, 1.0 to 4.5, describe patients with a high degree of ambulatory ability, and the following levels, 5.0 to 9.5, designate a gradual loss of mobility. An inclusion criterion of a EDSS value <4 confirms that all patients have the physical capability to perform the pelvic floor training program. Females were excluded from the study if their EDSS score rose by more than 0.5 points from their starting point.

All women were assessed using the Female Sexual Function Index (FSFI) and Female Sexual Distress Scale-Revised (FSDS-R) at the initiation and conclusion of this study. To ensure the reliability and accuracy of the FSFI and FSDS-R results, a pre-study training session was held for healthcare professionals who assisted the participants. This training aimed to standardise the guidance provided to participants during the questionnaire completion. The sexual function of the patients was evaluated using the Female Sexual Function Index (FSFI) questionnaire. This comprises 19 self-report questions and classifies sexual dysfunction into six domains, namely desire, arousal, lubrication, orgasm, satisfaction and pain [18]. To calculate individual domain scores, the results of specific items within each domain were added and multiplied by the domain factor. The full-scale score was determined by adding the scores of all six domains. A higher score indicates a better result. Participants were classified as likely to have sexual dysfunction when the total FSFI score was less than 26.5. This study used a questionnaire, which had been translated into the Greek language and validated [19]. Participants completed the questionnaire during their assessment visits, with the assistance of a health care professional allowed if there was any doubt or need for the clarification of questions.

The Female Sexual Distress Scale-Revised (FSDS-R) was created and validated to assess sex-related distress associated with inadequate or impaired sexual function [20]. It is a potentially useful adjunct to other validated sexual function questionnaires because it allows an assessment of distress related to sexual function. Using the FSDS, it was found that 44% of women reported a sexual complaint, but only 12% reported “distress” with sexual function. These data helped to create a structure of reference and evaluate the real impact of sexual complaints on quality of life. A score of 11 effectively discriminates between women with FSD and no FSD. The FSDS-R questionnaire was translated into the Greek language, but it was not validated.

The pelvic floor muscle training consisted of three office biofeedback sessions and home therapy. The nurses conducting the training were periodically evaluated to ensure the quality and consistency of the training provided. The home exercise regimen consisted of a progressive increase in muscle contractions. The exercise regimen comprised five rapid and ten sustained contractions with a ten-second rest interval in between. Over time, the number of sustained contractions was progressively increased, culminating in a final regimen of five rapid and twenty sustained contractions performed twice daily. PFMT implementation was supervised by trained nurses, as was assessment. All participants maintained a regular schedule of weekly office visits for a period of 12 weeks. Monthly 30-min biofeedback sessions, along with regular contact with registered nurses, supplemented training. No electromyogram (EMG) evaluation was undertaken during PFMT.

The realisation that many participants were incorrectly performing PFMT emphasised the necessity for individual training under the guidance of a skilled practitioner. For all participants, the assessment f pelvic floor muscle contraction capability involved vaginal palpation and observation of inward movement.

Descriptive statistics such as mean, standard deviation and percentage values were utilised to summarise variables. Chi-square was used for the comparison of categorical variables between the two groups (PFMT versus Control group). A value of p less than 0.05 was considered statistically significant. All data were anonymised and handled in accordance with GDPR guidelines. This study adhered to CONSORT guidelines. Data analysis was undertaken using the SPSS (IBM, Armonk, NY, USA, Version 23) statistical package program.

## 3. Results

The present study entailed the participation of eighty-two MS females. They were equally divided into two groups. Four women from Group A and three from Group B refused to fill out the questionnaires at the end of the trial and were, therefore, excluded. Three females from Group A did not continue PFMT because of fatigue or difficulty performing pelvic floor exercises during the day. One woman from Group A and two from Group B presented with blurred vision during this study and started treatment with a new regimen. These unforeseen medical complications underscore the dynamic nature of MS and its unpredictable impact on participants’ ability to fully engage in this study. Following all these exclusions, there were 33 women in Group A and 36 in Group B who completed this study.

At the outset, women with MS exhibited weakness during manual muscle testing and had limited short pelvic floor muscle endurance. Only eight patients among the women demonstrated more pronounced bladder neck elevation. This initial evaluation was crucial for customizing the PFMT to the individual’s physical capabilities. The overall mean age of the patient population was 38.4 ± 5.2, while the average age at which the disease was diagnosed was 31.1 ± 2.9 years. Regarding demographic data, there were no significant differences observed between the two groups (*p* > 0.05) (Table 2). All women received an interferon b regimen for MS treatment, and no one discontinued treatment during protocol.

Differences in baseline sexual function and distress variables were insignificant between groups A and B (*p* > 0.05) (Table 3). Following the twelve-week period, Group A demonstrated a statistically significant improvement in almost all domains of FSFI and FSDS-R compared to Group B (Table 3).

Women with PFMT (Group A) displayed lower statistically significant results (*p* < 0.05) for their FSDS-R scores (7.23 ± 3.72 vs. 9.35 ± 4.41). There was no statistically significant difference in the pain domain. Interestingly, the lack of significant change in the pain domain suggests that while PFMT can enhance sexual function, it may not directly impact sexual pain. There was no report of important harm from or unintended effects of PFMT in women of Group A. This is a reassuring indication of the safety and tolerability of PFMT for this patient population. By the end of the trial period, twelve Group A women reported that their sexual difficulties were resolved, while no women in Group B reported the same outcome. This disparity not only underscores the potential benefits of PFMT but also emphasises the necessity of using targeted interventions for sexual dysfunction in MS.

## 4. Discussion

A 14,268 MS patients survey by the North American Research Committee on Multiple Sclerosis (NARCOMS) reported that one-third of MS patients experienced moderate-to-severe pelvic floor symptoms (bladder, 41%; bowel, 30%; sexual, 42%). These symptoms have a negative effect on routine activities, walking and quality of life in physical terms [21].

There are a lack of data on the treatment of SD in women with MS. The results of sildenafil use in MS women have been contradictory [22]. Tricyclic antidepressants, anticonvulsants and topical local anaesthetics can alleviate hypersensitivity in the genitalia of female MS patients [23]. Water-soluble vaginal lubricants and oestrogen combined with methyltestosterone increase vaginal lubrication and heighten sexual desire in MS females with reduced lubrication [24]. Furthermore, recent investigations into neuromuscular electrical stimulation and PFMT have shown promising results for improving SD and urinary symptoms in MS patients [25].

Alternatives to sexual activity, like the use of a vibrator, may be taken into consideration for women with MS. Both non-pharmacological and pharmaceutical treatments are used for fatigue. Sexual activity earlier in the day should be encouraged because energy levels decrease in the afternoon and evening [24]. Anti-fatigue medications are an alternative option [23]. To improve sexual function, patients dealing with spasticity may find it advantageous to adopt more comfortable positions, employing pillows, and/or engage in muscle-relaxing treatments (such as massage and stretching). As an alternative, individuals can take medications such baclofen, tizanidine or benzodiazepines before engaging in sexual activity [26]. Furthermore, treating bladder dysfunction enhances sexual function in MS patients [26]. For those performing clean intermittent self-catheterisation, there is a recommendation to empty the bladder prior to sexual activity.

According to this study’s findings, the sexual function of MS women who underwent PFMT significantly improved. A study by Lúcio et al. supports the beneficial impact of PFMT, both alone and in combination with other treatments like intravaginal NMES or TTNS, in improving SD, suggesting its adaptability to individual patient needs [27]. Women who were not neurologically impaired but suffered from stress urinary incontinence and had PFMT as a treatment reported an unanticipated improvement in their sexual response [28]. PF rehabilitation has shown improvements in the severity of lower urinary tract symptoms, quality of life, level of anxiety and depression and sexual dysfunction from MS treatment [29]. Certain authors have proposed that PFMT, be it alone or in combination with other stimulation techniques, can serve as a beneficial approach in treating sexual dysfunction among MS patients [27]. There are several models explaining the constructive role of PFMT on sexual function. The structure of pelvic floor muscles presents several changes after a PFMT program. Enhanced cross-sectional diameter may potentially improve the vaginal friction sensation for women during intercourse and increase the sensation of vaginal grip experienced by her partner. Moreover, the improvement in neuromuscular function can be attributed to the increase in the number of activated motor neurons and improved circulation to the genital organ. Strengthening PFM is associated with better muscle performance during orgasmic PFM rhythmic contractions. It also plays a role in controlling the anatomical position of the clitoris during erectile stages and facilitates enhanced sexual stimulation [30,31].

The potential for PFMT to improve sexual function is further supported by Gopal et al., who found that various physical therapy interventions, including PFMT, significantly improved sexual function, satisfaction and emotional well-being in individuals with MS [32]. Our results are not in accordance with research presented by Mosalanejad et al. [16]. In this clinical trial, mindfulness and PFMT did not have any notable impacts on the sexual dysfunction of women suffering from MS. All study participants expressed positive acceptance of PFMT, as it was considered easy to implement and free from adverse effects. However, further study is required to make conclusive clinical judgements regarding the effectiveness of these interventions.

Several authors have reported a noteworthy correlation between incontinence and SD, with both disorders associated with the degree of neurological impairment. Additionally, evidence from a study by Hwang et al. (2019) indicates that improvements in pelvic floor muscle parameters like strength, power and endurance are associated with better sexual function, emphasizing the interconnectedness between physical and sexual health [33]. To strengthen our research, the women formed a convenient sample of MS patients without incontinence problems and low disability (mean ESSS scale 2.1–2.3 and <4). These factors highlight the significance of early intervention, particularly prior to the onset of the disability. Therefore, when sexual issues are identified through patient evaluations in the early diagnosis of MS, PFMT emerges as an easy-to-apply exercise that patients can engage in, even before the manifestations of symptoms.

The therapeutic use of IFNs has been linked to an elevated susceptibility to subjective sensations of illness, such as fatigue and depression, collectively recognised as cytokine sickness behaviour [34]. Although the cautionary labels for all MS IFN β therapies acknowledge the potential for depression and suicide, much of the available literature does not support the connection between IFN β therapy and depression. A systematic review encompassing 10 studies indicated no substantial correlation between depression levels and IFN treatment. While the majority did not propose an association between depression and IFN β therapy, three studies suggested that individuals with a history of depression might experience a major depressive episode within the first 6 months of treatment [35].

Together with the neurological symptoms that characterise MS, depression is also typically observed, with estimates indicating a lifetime prevalence as high as 60% following diagnosis [36]. Previous findings indicate that even the patients who independently engage in PFMT experience improvements in their symptoms of depression. Therefore, in order to enhance the quality of life for those with MS, further research on the pelvic floor function and the development of additional methods to promote exercise are necessary [30,37]. A recent review by Bahmani and Motl highlighted the positive effect of physical exercise on SD in people with MS. The authors proposed that consistent engagement in physical activity leads to a decrease in symptoms of depression and the severity of fatigue. It is also associated with increased self-esteem, decreased pain perception and improved sleep quality. Consequently, it has the potential to positively impact the secondary and tertiary aspects of SD. Furthermore, exercise-induced neurophysiological changes may promote sex drive and sexual satisfaction [38].

An assessment of cultural, psychological or social aspects that can impact SD was not undertaken and that is a limitation of our study. Study participants had EDSS scores of 4.0 or lower. This suggests that the effectiveness of PFMT may be associated with mild levels of disability. The effectiveness of PFMT in patients with high EDSS scores remains a topic of debate. Furthermore, a UK consensus recommends that “pelvic floor exercises should be offered to patients with mild disability from MS”, emphasising the importance of assessing pelvic floor contractions prior to treatment initiation [39]. The present study evaluated the short-term effects of PFMT over a twelve-week period. However, the long-term effects should also be studied. Despite the long-standing application of pelvic floor rehabilitation in the treatment of MS, there remains no standardised protocol for pelvic floor dysfunction, as regards PFMT duration or approach to treatment [29]. Another limitation of this study is that the FSDS questionnaire is only translated into Greek but still needs to be validated.

Sexuality has been recognised as a very sensitive issue in almost every culture [4]. Τhe majority of people hesitate to discuss their sexual problems and intimacy. The proportions of MS patients who were willing to talk about sexual problems with doctors and friends are very low for women (ranging from 3.0 to 7.4%), without significant increase over the 6-year period [9]. However, there was a significant increase in the likelihood of discussing sexual issues with partners, rising from 22.7% at baseline to 30.3% for female participants. Furthermore, it is important to note that healthcare professionals encountered various barriers to addressing sexual dysfunction in their patients [40]. The most commonly reported challenges included personal embarrassment, time constrains, inadequate training and concerns regarding their ability to effectively address the raised issues [41].

Moreover, the subjective experience of sexual dysfunction in women with MS suggests that individual perception and coping mechanisms significantly influence the overall impact on quality of life and relationship dynamics. This is evident from the variance in responses to PFMT and other interventions across studies. For instance, a study by Dunya et al. evaluated the effects of transcutaneous tibial nerve stimulation and PFMT on sexual dysfunction in female MS patients and reported improvements in sexual function, emphasizing the need for personalised treatment plans [25].

It is also important to consider the psychological and social aspects of MS and its treatment. While physical interventions like PFMT can offer significant benefits, comprehensive care that includes psychological support and social counseling may further enhance outcomes. A holistic approach to treatment, considering not just the physical but also the emotional and social well-being of the patient, could provide a more robust and supportive environment for managing sexual dysfunction in women with MS.

In conclusion, while PFMT and related physical therapies show promise in addressing sexual dysfunction in MS patients, further research is needed to understand the full spectrum of factors affecting sexual health in this population. Personalised treatment plans, a holistic approach to care and a deeper understanding of the individual’s experience are crucial for improving quality of life for women with MS.

## 5. Conclusions

This study indicates that PFMT contributes to the improvement of FSD in women suffering from MS. Participants in this study displayed significant enhancements in desire, arousal, vaginal lubrication and overall sexual satisfaction, as evidenced by improvements in the total scores of the FSFI and FSDD-R questionnaire. The findings suggest that PFMT, as a therapeutic intervention, not only bolsters sexual function but also attenuates sexual distress, contributing to a markedly improved quality of life for women with MS. These promising results advocate for a more prominent role of PFMT in the management of FSD, particularly among MS women, reaffirming the critical need for holistic, patient-centred care approaches that address the comprehensive well-being of individuals affected by this condition.

## Figures and Tables

**Table 1:** Inclusion and exclusion criteria for this study on the effects of pelvic floor muscle training in women with multiple sclerosis.

Inclusion Criteria	Exclusion Criteria
18 years of age or older	Previous pelvic floor muscle training program
Diagnosis of relapsing–remitting MS	Ongoing pregnancy
Stable condition for a minimum period of six months	Child delivery within the previous six months
Kurtzke’s Expanded Disability Status Scale (EDSS) score < 4	Urinary or faecal incontinence
Women sexually active for at least four weeks	Pelvic organ prolapses greater than stage I
Cognitive ability to complete the questionnaires and study protocol	Perimenopause or menopause period
Ability to contract PFM evaluated by the primary investigator	

**Table 2:** Demographic data of the two groups of this study presented as mean value ± SD (standard deviation).

Variables	Groups		*p* Value
	PFMT Group A, 33 pts	Control Group B, 36 pts	
Mean Age (years) ± SD	38.1 ± 3.2	39.3 ± 5.1	*p* > 0.05
Mean Duration of MS (years) ± SD	8.1 ± 2.6	7.5 ± 2.3	*p* > 0.05
Mean Age of MS Diagnosis (years) ± SD	32.0 ± 2.5	30.8 ± 2.9	*p* > 0.05
Mean EDSS ± SD	2.3 ± 0.9	2.1 ± 1.0	*p* > 0.05

**Table 3:** FSFI and FSDS-R questionnaires’ data at the beginning and end of this study presented as mean value ± SD (standard deviation).

Domain	Group A	Group B	*p* Value
Patients’ evaluation at the beginning of this study			
**Desire**	2.97 ± 0.2	2.85 ± 0.29	*p* > 0.05
**Arousal**	3.91 ± 0.49	3.81 ± 0.41	*p* > 0.05
**Lubrication**	3.67 ± 0.40	3.78 ± 0.48	*p* > 0.05
**Orgasm**	3.79 ± 0.39	3.90 ± 0.35	*p* > 0.05
**Satisfaction**	3.76 ± 0.35	3.92 ± 0.37	*p* > 0.05
**Pain**	4.07 ± 0.44	4.26 ± 0.42	*p* > 0.05
**Total FSFI score**	22.17 ± 1.74	22.52 ± 1.93	*p* > 0.05
**FSDS-R**	9.62 ± 4.37	9.44 ± 4.01	*p* > 0.05
Patients’ evaluation at the end of this study			
**Desire**	3.54 ± 0.28	2.94 ± 0.25	*p* = 0.02
**Arousal**	4.33 ± 0.49	3.92 ± 0.47	*p* = 0.03
**Lubrication**	4.81 ± 0.46	3.82 ± 0.44	*p* = 0.04
**Orgasm**	4.91 ± 0.57	3.93 ± 0.38	*p* = 0.03
**Satisfaction**	5.01 ± 0.56	3.96 ± 0.39	*p* = 0.01
**Pain**	4.42 ± 0.42	4.31 ± 0.41	*p* > 0.05
**Total FSFI score**	27.02 ± 1.87	22.88 ± 1.81	*p* = 0.04
**FSDS-R**	7.23 ± 3.72	9.35 ± 4.51	*p* = 0.03

## Data Availability

The datasets used and/or analysed during the current study are available from the corresponding author on reasonable request.

## References

[1] Laumann E.O., Paik A., Rosen R.C. (1999). Sexual dysfunction in the United States: Prevalence and predictors. JAMA.

[2] Stephenson K.R., Meston C.M. (2015). Why is impaired sexual function distressing to women? The primacy of pleasure in female sexual dysfunction. J. Sex. Med..

[3] Khajehei M., Doherty M., Tilley P.J. (2015). An update on sexual function and dysfunction in women. Arch. Womens Ment. Health.

[4] Drulovic J., Kisic-Tepavcevic D., Pekmezovic T. (2020). Epidemiology, diagnosis and management of sexual dysfunction in multiple sclerosis. Acta Neurol. Belg..

[5] Schairer L.C., Foley F.W., Zemon V., Tyry T., Campagnolo D., Marrie R.A., Gromisch E.S., Schairer D. (2014). The impact of sexual dysfunction on health-related quality of life in people with multiple sclerosis. Mult. Scler..

[6] Yazdani A., Ebrahimi N., Mirmosayyeb O., Ghajarzadeh M. (2023). Prevalence and risk of developing sexual dysfunction in women with multiple sclerosis (MS): A systematic review and meta-analysis. BMC Womens Health.

[7] Bronner G., Elran E., Golomb J., Korczyn A.D. (2010). Female sexuality in multiple sclerosis: The multidimensional nature of the problem and the intervention. Acta Neurol. Scand..

[8] Tzortzis V., Skriapas K., Hadjigeorgiou G., Mitsogiannis I., Aggelakis K., Gravas S., Poulakis V., Melekos M.D. (2008). Sexual dysfunction in newly diagnosed multiple sclerosis women. Mult. Scler..

[9] Kisic-Tepavcevic D., Pekmezovic T., Trajkovic G., Stojsavljevic N., Dujmovic I., Mesaros S., Drulovic J. (2015). Sexual dysfunction in multiple sclerosis: A 6-year follow-up study. J. Neurol. Sci..

[10] DasGupta R., Fowler C.J. (2003). Bladder, bowel and sexual dysfunction in multiple sclerosis: Management strategies. Drugs.

[11] Dehghan-Nayeri N., Khakbazan Z., Ghafoori F., Nabavi S.M. (2017). Sexual dysfunction levels in iranian women suffering from multiple sclerosis. Mult. Scler. Relat. Disord..

[12] Zamani M., Tavoli A., Yazd Khasti B., Sedighimornani N., Zafar M. (2017). Sexual Therapy for Women with Multiple Sclerosis and Its Impact on Quality of Life. Iran. J. Psychiatry.

[13] Mahajan S.T., James R., Frasure H. (2014). Pelvic floor disorders and multiple sclerosis: Are patients satisfied with their care?. Int. J. MS Care.

[14] Omodei M.S., Marques Gomes Delmanto L.R., Carvalho-Pessoa E., Schmitt E.B., Nahas G.P., Petri Nahas E.A. (2019). Association Between Pelvic Floor Muscle Strength and Sexual Function in Postmenopausal Women. J. Sex. Med..

[15] Sapouna V., Thanopoulou S., Papriakas D., Papakosta S., Sakopoulou M., Zachariou D., Zikopoulos A., Kaltsas A., Vrachnis N., Vrachnis D. (2023). Pelvic Floor Muscle Training and Its Benefits for Multiple Sclerosis Patients Suffering From Urinary Incontinence and Sexual Dysfunction. Cureus.

[16] Mosalanejad F., Afrasiabifar A., Zoladl M. (2018). Investigating the combined effect of pelvic floor muscle exercise and mindfulness on sexual function in women with multiple sclerosis: A randomized controlled trial. Clin. Rehabil..

[17] Giannopapas V., Kitsos D., Tsogka A., Tzartos J.S., Paraskevas G., Tsivgoulis G., Voumvourakis K., Giannopoulos S., Bakalidou D. (2023). Sexual dysfunction therapeutic approaches in patients with multiple sclerosis: A systematic review. Neurol. Sci..

[18] Rosen R., Brown C., Heiman J., Leiblum S., Meston C., Shabsigh R., Ferguson D., D’Agostino R. (2000). The Female Sexual Function Index (FSFI): A multidimensional self-report instrument for the assessment of female sexual function. J. Sex Marital Ther..

[19] Zachariou A., Filiponi M., Kirana P.S. (2017). Translation and validation of the Greek version of the female sexual function index questionnaire. Int. J. Impot. Res..

[20] Derogatis L., Clayton A., Lewis-D’Agostino D., Wunderlich G., Fu Y. (2008). Validation of the female sexual distress scale-revised for assessing distress in women with hypoactive sexual desire disorder. J. Sex. Med..

[21] Aguilar-Zafra S., Del Corral T., Vidal-Quevedo C., Rodriguez-Duran P., Lopez-de-Uralde-Villanueva I. (2020). Pelvic floor dysfunction negatively impacts general functional performance in patients with multiple sclerosis. Neurourol. Urodyn..

[22] Dasgupta R., Wiseman O.J., Kanabar G., Fowler C.J., Mikol D. (2004). Efficacy of sildenafil in the treatment of female sexual dysfunction due to multiple sclerosis. J. Urol..

[23] Delaney K.E., Donovan J. (2017). Multiple sclerosis and sexual dysfunction: A need for further education and interdisciplinary care. NeuroRehabilitation.

[24] Orasanu B., Frasure H., Wyman A., Mahajan S.T. (2013). Sexual dysfunction in patients with multiple sclerosis. Mult. Scler. Relat. Disord..

[25] Polat Dunya C., Tulek Z., Kurtuncu M., Gunduz T., Panicker J.N., Eraksoy M. (2021). Evaluating the effects of transcutaneous tibial nerve stimulation or pelvic floor muscle training on sexual dysfunction in female multiple sclerosis patients reporting overactive bladder. Neurourol. Urodyn..

[26] Drulovic J., Pekmezovic T., Matejic B., Mesaros S., Manigoda M., Dujmovic I., Stojsavljevic N., Kocev N., Gavric-Kezic M., Nikic P. (2007). Quality of life in patients with multiple sclerosis in Serbia. Acta Neurol. Scand..

[27] Lucio A.C., D’Ancona C.A., Lopes M.H., Perissinotto M.C., Damasceno B.P. (2014). The effect of pelvic floor muscle training alone or in combination with electrostimulation in the treatment of sexual dysfunction in women with multiple sclerosis. Mult. Scler..

[28] Zahariou A.G., Karamouti M.V., Papaioannou P.D. (2008). Pelvic floor muscle training improves sexual function of women with stress urinary incontinence. Int. Urogynecol. J. Pelvic Floor Dysfunct..

[29] Sparaco M., Bonavita S. (2022). Pelvic Floor Dysfunctions and Their Rehabilitation in Multiple Sclerosis. J. Clin. Med..

[30] Altunan B., Gundogdu A.A., Ozcaglayan T.I.K., Unal A., Turgut N. (2021). The effect of pelvic floor exercise program on incontinence and sexual dysfunction in multiple sclerosis patients. Int. Urol. Nephrol..

[31] Lowenstein L., Gruenwald I., Gartman I., Vardi Y. (2010). Can stronger pelvic muscle floor improve sexual function?. Int. Urogynecol. J..

[32] Gopal A., Sydow R., Block V., Allen D.D. (2021). Effectiveness of Physical Therapy in Addressing Sexual Dysfunction in Individuals with Multiple Sclerosis: A Systematic Review and Meta-analysis. Int. J. MS Care.

[33] Hwang U.J., Lee M.S., Jung S.H., Ahn S.H., Kwon O.Y. (2019). Pelvic Floor Muscle Parameters Affect Sexual Function After 8 Weeks of Transcutaneous Electrical Stimulation in Women with Stress Urinary Incontinence. Sex. Med..

[34] Dantzer R. (2009). Cytokine, sickness behavior, and depression. Immunol. Allergy Clin. N. Am..

[35] Alba Pale L., Leon Caballero J., Samso Buxareu B., Salgado Serrano P., Perez Sola V. (2017). Systematic review of depression in patients with multiple sclerosis and its relationship to interferonbeta treatment. Mult. Scler. Relat. Disord..

[36] Persson R., Lee S., Yood M.U., Wagner M.R., Minton N., Niemcryk S., Lindholm A., Evans A.M., Jick S. (2020). Incident depression in patients diagnosed with multiple sclerosis: A multi-database study. Eur. J. Neurol..

[37] Ferreira A.P., Pegorare A.B., Salgado P.R., Casafus F.S., Christofoletti G. (2016). Impact of a Pelvic Floor Training Program Among Women with Multiple Sclerosis: A Controlled Clinical Trial. Am. J. Phys. Med. Rehabil..

[38] Sadeghi Bahmani D., Motl R.W. (2021). Rate, burden, and treatment of sexual dysfunction in multiple sclerosis: The case for exercise training as a new treatment approach. Mult. Scler. Relat. Disord..

[39] Lensch E., Jost W.H. (2011). Autonomic disorders in multiple sclerosis. Autoimmune Dis..

[40] Gott M., Galena E., Hinchliff S., Elford H. (2004). “Opening a can of worms”: GP and practice nurse barriers to talking about sexual health in primary care. Fam. Pract..

[41] Proietti S., Giannantoni A., Sahai A., Khan M.S., Dasgupta P. (2012). Overactive bladder and sexual function: A nightmare couple. BJU Int..

